# A longitudinal community-based ototoxicity monitoring programme and treatment effects for drug-resistant tuberculosis treatment, Western Cape

**DOI:** 10.4102/sajcd.v69i1.886

**Published:** 2022-03-31

**Authors:** Lucia J. Stevenson, Leigh Biagio-de Jager, Marien A. Graham, De Wet Swanepoel

**Affiliations:** 1 Department of Speech-Language Pathology and Audiology, Faculty of Humanities, University of Pretoria, Pretoria, South Africa; 2 Department of Science, Mathematics and Technology Education, Faculty of Education, University of Pretoria, Pretoria, South Africa; 3 Ear Science Institute Australia, Perth, Australia

**Keywords:** community-based services, community health workers, decentralised services, tuberculosis, drug-resistant tuberculosis, hearing loss, ototoxicity monitoring, audiometry, South Africa

## Abstract

**Background:**

South Africa has a high burden of drug-resistant tuberculosis (DRTB) and until recently, ototoxic aminoglycosides were predominant in treatment regimens. Community-based ototoxicity monitoring programmes (OMPs) have been implemented for early detection of hearing loss and increased patient access.

**Objectives:**

A longitudinal study was conducted to describe the service delivery characteristics of a community-based OMP for DRTB patients facilitated by CHWs as well as observed ototoxic hearing loss in this population.

**Method:**

A descriptive retrospective record review of longitudinal ototoxicity monitoring of 194 DRTB patients undergoing treatment at community-based clinics in the city of Cape Town between 2013 and 2017.

**Results:**

Follow-up rates between consecutive monitoring assessments reached as high as 80.6% for patients assessed by CHWs. Few patients (14.2% – 32.6%) were assessed with the regularity (≥ 6 assessments) and frequency required for effective ototoxicity monitoring, with assessments conducted, on average, every 53.4–64.3 days. Following DRTB treatment, 51.5% of patients presented with a significant ototoxic shift meeting one or more of the American Speech-Language-Hearing Association (ASHA) criteria. Deterioration in hearing thresholds was bilateral and most pronounced at high frequencies (4 kHz – 8 kHz). The presence of pre-existing hearing loss, human immunodeficiency virus co-infection and a history of noise exposure were significant predictors of ototoxicity in patients.

**Conclusion:**

DRTB treatment with kanamycin resulted in significant deterioration of hearing longitudinally, predominantly at high frequencies. With ongoing training and supportive supervision, CHWs can facilitate community-based ototoxicity monitoring of DRTB patients. Current protocols and guidelines may require reassessment for appropriate community-based ototoxicity monitoring.

## Introduction

Tuberculosis (TB) is a communicable disease spread when people who are sick expel the TB-causing bacteria into the air (World Health Organisation [WHO], 2020a). Although TB can be successfully prevented and treated (Cox et al., 2019), it is the leading infectious disease and one of the top 10 causes of death globally (WHO, 2020a). Africa accounted for 25% of the 10 million people globally who developed TB in 2019, with South Africa being identified by the WHO as a high-burden TB country (WHO, 2020a). Furthermore, South Africa has a high prevalence rate (19.5%) of human immunodeficiency virus (HIV) infection for adults aged 15–49 years (Republic of South Africa Statistics Department, 2021; Wells et al., 2007). In 2019, 209 000 people in the country were afflicted with TB and HIV (WHO, 2020a). Tuberculosis may accelerate the course of HIV infection, which may contribute to the increase in the prevalence of drug-resistant TB (DRTB) in patients with TB (Wells et al., 2007).

Tuberculosis that is resistant to at least two of the most effective anti-TB drugs, rifampicin and isoniazid, is known as DRTB (Centers for Disease Control and Prevention [CDC], 2016). Rifampicin-resistant TB (RRTB) and multidrug-resistant TB (MDRTB), which are different types of DRTB, continue to be a public health threat (WHO, 2020a) that jeopardises the control of TB (Horsburgh, Mitnick, & Lange, 2019). Close to half a million people developed RRTB globally in 2019, 78% of whom had MDRTB (WHO, 2020a). South Africa has the highest number of patients with MDRTB on the African continent (Lange et al., 2019), with an estimated 23 out of every 100 000 people being infected with RR/MDRTB (WHO, 2020a).

Treatment of DRTB takes longer and requires drugs that are more expensive and more toxic than those used for the treatment of TB (WHO, 2020a). Before 2018, the WHO and the South African Department of Health (Department Health Republic of South Africa [DOH], 2013) included the use of a second-line injectable antibiotic (either an aminoglycoside such as kanamycin, or a polypeptide) in the DRTB treatment regimen (Wrohan, Redwood, Ho, Velen, & Fox, 2021). Aminoglycoside antibiotics are known to affect hearing and balance, or both, through ototoxicity in the cochleovestibular organ (Campbell & Le Prell, 2018). Outer hair cell damage starts at the basal coil and progresses to the apex of the cochlea, resulting in a permanent high-frequency hearing loss, progressing to the lower frequencies (De Jager & Van Altena, 2002). Damage to the outer hair cells is followed by progressive loss of the inner hair cells in more severe cases (Xie, Talaska, & Schacht, 2011). The prevalence rate of aminoglycoside-induced ototoxicity in DRTB patients, which is estimated as 63% of patients (WHO, 2021b), is dependent on the drug, drug dosage, treatment duration (Huth, Ricci, Cheng, & Pearson, 2011; Schacht, Talaska, & Rybak, 2012; Xie et al., 2011) and patients’ demographic profile (Ramma, Heinze, & Schellack, 2019). In the Western Cape, it has been reported that around 47% – 57% of DRTB patients have developed aminoglycoside-induced hearing loss (Melchionda et al., 2013; Petersen & Rogers, 2015; Ramma et al., 2019).

Concerns regarding the ototoxic nature of injectable antibiotics and the availability of novel, less toxic, more effective drugs led to an update of the South African Department of Health (DOH, 2018) and WHO DRTB treatment guidelines in 2018 and 2019, respectively (WHO, 2020b; Wrohan et al., 2021). The latest DRTB treatment guidelines recommend a shorter, all-oral regimen containing bedaquiline for the treatment of RR/MDRTB (DOH, 2018; WHO, 2020b). Bedaquiline has fewer side effects than the other drugs used to treat DRTB (Medicins Sans Frontieres [MSF], 2020) and does not appear to be associated with hearing loss, unlike kanamycin (Khoza-Shangase & Prodromos, 2021). However, an all-oral regimen may not be suitable for all patients, and therefore the guidelines continue to include the use of amikacin, which is associated with an estimated hearing loss prevalence of 38.9% (Dillard et al., 2021; Evans et al., 2015; WHO, 2020b; Wrohan et al., 2021). Furthermore, access to novel drugs has remained limited (MSF, 2020). Between 2015 and 2019, only one in nine people across 36 countries who could benefit from bedaquiline received the medication (Cox et al., 2018; MSF, 2020). Despite their adverse effects, aminoglycoside antibiotics are used in high-burden TB countries because they are easily accessible and inexpensive, leading to an increased burden of aminoglycoside induced hearing loss (Bardien et al., 2009; Campbell & Le Prell, 2018). Almost half (46%; 17/37) of the countries whose national policies and practices were surveyed in 2019 (MSF, 2020) reported still using kanamycin or capreomycin in the treatment of DRTB, contrary to the latest recommendations (WHO, 2020a). In addition, the coronavirus disease 2019 (COVID-19) pandemic threatens to undo the progress made in TB control as it causes major disruptions to essential TB services and threatens to increase the burden of TB disease (WHO, 2020a). As a result, a substantial number of patients may develop ototoxic hearing loss and require hearing loss prevention strategies, including audiological ototoxicity monitoring (Dillard et al., 2021).

When the use of injectable ototoxic medications is unavoidable, audiological ototoxicity monitoring is essential to optimise hearing-related outcomes (WHO, 2021b; Wrohan et al., 2021). Audiological ototoxicity monitoring encompasses the regular assessment of patients’ hearing thresholds during treatment to detect early changes in hearing, so that treatment regimens can be adjusted and disabling hearing loss can be avoided (WHO, 2021b). In response to the high prevalence of ototoxic hearing loss associated with DRTB treatment, the South African National TB Control Programme implemented the National Ototoxicity Prevention Programme to improve the access to audiological monitoring and reduce the prevalence of ototoxic hearing loss (WHO, 2021b). As part of this programme, portable audiometers and training were offered to selected decentralised health facilities, including primary healthcare (PHC) facilities (WHO, 2021b). Patients with DRTB were able to access ototoxicity monitoring services outside centralised TB hospitals, increasing access to care (DOH, 2013; Ndjeka et al., 2020; The South African National Aids Council, 2017).

The South African Department of Health has committed to addressing the disparity in human resources for health by prioritising the integration of 50 000 community health workers (CHWs) into the PHC system by 2024 (DOH, 2020). Community health workers are individuals working in the community in which they reside who are selected and trained to broaden the access and coverage of health care services in remote areas (WHO, 2007). Community health workers engage in task-sharing, which involves the shifting of health care tasks from highly skilled professionals such as audiologists to workers with shorter training, such as CHWs (Dillard et al., 2021; DOH, 2020). Task shifting (Mulwafu, Ensink, Kuper, & Fagan, 2017) and incorporating ototoxicity monitoring into existing service delivery models, such as community-based health care services, have been proposed to address the barriers to ototoxicity monitoring (Dillard et al., 2021).

To improve the efficacy and efficiency for early detection of hearing changes, existing ototoxicity monitoring programmes (OMPs) and treatment effects should be evaluated so that ototoxicity monitoring guidelines can be adapted to specific settings (Dillard et al., 2021; Health Professions Council of South Africa [HPCSA], 2018). The current study, therefore, aimed to describe the service delivery characteristics of a community-based OMP for patients with DRTB, facilitated by CHWs and PHC audiologists using conventional audiometry (0.25 kHz – 8 kHz) for ototoxicity monitoring according to the timing, frequency and follow-up rates of ototoxicity monitoring assessments. In addition, this study aimed to describe the ototoxic hearing loss observed in DRTB patients over time. To our knowledge, this is the first study to report on observed longitudinal treatment effects for DRTB and ototoxicity monitoring conducted by CHWs in a decentralised community-based model of care for increased patient access.

## Materials and method

This study was part of a larger, longitudinal descriptive retrospective record review of a decentralised community-based OMP for patients with DRTB facilitated by CHWs between 2013 and 2017. This specific OMP was selected for investigation as it offers a novel approach to ototoxicity monitoring for DRTB patients with a timeframe allowing for as many study participants as possible. The data were collected at community health centres and PHC clinics in two sub-districts of the city of Cape Town (CoCT), namely the [location masked for blind review] and [location masked for blind review] sub-districts. At the time of data collection, the sub-districts were characterised by a predominantly coloured (30% – 50%) and black African (19% – 46%) population who mostly resided in formal dwellings (68% – 87%) (CoCT, 2013a, 2013b, 2013c, 2013d). Most people living in the sub-districts included in this study were employed (68% – 87%), with 32% – 60% of people having completed high school education (Grade 12) (CoCT, 2013a, 2013b, 2013c, 2013d). This study aimed to supplement the findings of a larger study by describing the service delivery characteristics of a community-based OMP for DRTB patients facilitated by CHWs and PHC audiologists and the ototoxic hearing loss observed in this population over time.

### Participants

**FIGURE 1:**
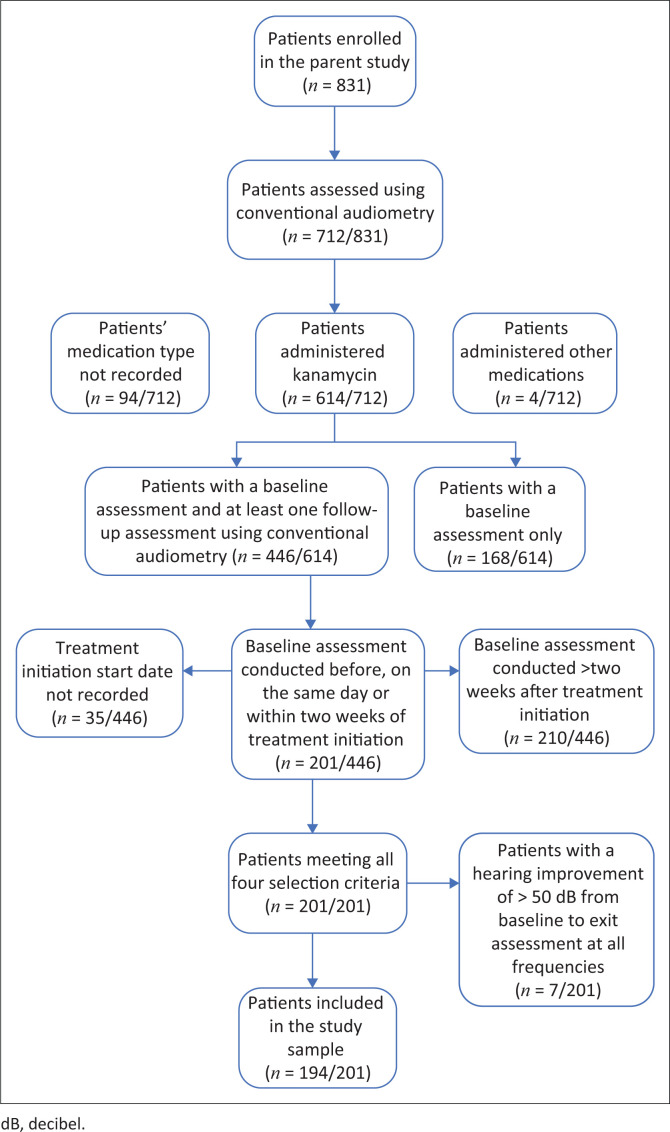
Patient selection procedure.

This study included patients from a larger study who met the following selection criteria: patients who (1) received kanamycin, (2) were tested using conventional audiometry (0.25 kHz – 8 kHz), (3) had a baseline assessment conducted before, on the same day, or within 2 weeks of initiation of medication, and (4) had one or more follow-up monitoring assessments using conventional audiometry thereafter. The selection criteria were based on the OMP protocol and guidelines for ototoxicity monitoring (HPCSA, 2018) to allow for comparability. Non-probability purposive sampling was used to select all patients with DRTB, regardless of age, gender or hearing status. Of the 831 patients included in the parent study, 194 patients met all the selection criteria and were eligible for inclusion in this study (Figure 1). The patient interviews and ototoxicity monitoring assessments were conducted by six CHWs and two PHC audiologists at 19 of the sub-districts’ PHC clinics and community health centres. In 2012, the Western Cape Department of Health, in collaboration with the University of Cape Town, initiated a pilot project in which 30 CHWs underwent a year-long certificate training programme to become members of the PHC team (Clark, 2015; Gamiet & Rowe, 2019). The CHWs were provided with skills and knowledge in community-based rehabilitation to support people with disabilities in two underserved communities in the Western Cape (Gamiet & Rowe, 2019). To facilitate ototoxicity monitoring for DRTB in a community-based setting, six CHWs received additional training from the PHC audiologist responsible for the Mitchells Plain/Klipfontein sub-district. The six CHWs were also trained to conduct home-based hearing screening and hearing screening of school-aged children and patients attending a PHC clinic.

### Data collection procedure

The data collection procedure for this study was the same as for the larger study (Stevenson et al., 2021). The OMP protocol implemented at the time of data collection was as follows: all patients who received ototoxic medication for treatment of DRTB were identified and referred by their managing doctor and included in the OMP as part of the package of care. Patients visited a clinic or centre daily for the first 6 months of treatment to receive their medication from a nurse. After the initial 6-month treatment period, medication was continued for 18 months, with patients visiting a clinic weekly to obtain their medication, and monthly to consult with their managing doctor. At the time of data collection, the official South African ototoxicity monitoring guidelines had not yet been published, thus the OMP developers relied on adapting the international guidelines of the American Speech-Language-Hearing Association (ASHA) (ASHA, 1994) and the American Academy of Audiology (AAA) (AAA, 2009) when developing the OMP protocol. An unpublished draft of the HPCSA ototoxicity monitoring guideline was, however, available to the OMP developers to assist them in applying the international guidelines to the South African context.

Community health workers and PHC audiologists travelled to the clinics in each sub-district with portable audiological equipment. Community health workers and PHC audiologists were testers in the Mitchells Plain/Klipfontein sub-district, whereas only PHC audiologists were testers in the Western or Southern sub-district. At the time of a patient’s baseline assessment, identifying information including the patient’s name, date of birth, gender and medical history pertaining to HIV status, DRTB medication/s, comorbidities and adverse effects were recorded manually on a paper data collection form by CHWs and PHC audiologists. This information was obtained from the patient’s medical records in a clinic file and/or verbally reported to the CHWs and PHC audiologists during the patient interview. The KUDUwave portable audiometer (eMoyo, South Africa) was used by CHWs and PHC audiologists in this study. The KUDU wave is a PC (Dell laptop) controlled clinical diagnostic audiometer, and integrated supra-aural ear-cup and insert earphone headset, with an electronic response button for use without a soundproof booth. Automated and manual programmes conduct audiometry up to 16 kHz. Results are stored electronically and store-and-forward for printing.

The protocol for baseline and monitoring audiological ototoxicity monitoring assessments followed by the OMP at the time of data collection was as follows: a bilateral otoscopic examination was conducted followed by air-conduction pure-tone audiometry, and the findings recorded on the data collection form. If outer or middle ear pathology was suspected following otoscopy, the patient was referred to the managing doctor or nurse for appropriate treatment and referred for audiometry, according to the OMP protocol. Baseline assessments were conducted at the clinics prior to, on the same day or within 2 weeks of DRTB treatment initiation. Monitoring assessments were conducted once a month during the initial 6-month treatment regimen and then at 3, 6 and 18 months thereafter. Where an ototoxic shift meeting predetermined criteria (ASHA, 1994) was evident, the managing doctor was informed, and monitoring assessments were then conducted every 2 weeks until no change in hearing thresholds was detected. Assessments were conducted in a quiet environment using conventional audiometry (0.25 kHz – 8 kHz). Typically, manual testing would have been done; however, an automatic mode of threshold determination may also have been used in some instances. The equipment required to conduct both conventional audiometry and extended high-frequency (EHF) audiometry became available in November 2015 for use in the [location masked for blind review], and in July 2016 for use in the [location masked for blind review] sub-district. Before this, only conventional audiometry was available for ototoxicity monitoring.

Each patient’s descriptive and audiological data were recorded manually by the CHWs and PHC audiologists on paper data collection forms and stored in the patient’s clinic file. A copy of each patient’s data collection form was kept with the CHWs and PHC audiologists and regularly made available for review to the managing PHC audiologist responsible for each sub-district. Upon completion of a patient’s DRTB treatment and ototoxicity monitoring, the form was stored permanently with the PHC audiologist responsible for each sub-district. The researchers collected the hardcopies of the patients’ data collection forms from the managing PHC audiologists in each sub-district for analysis, and these were returned upon completion of this study.

### Data analysis

Data were imported from Excel into Statistical Package for Social Sciences (SPSS) software (version 27), after which descriptive statistics such as frequency distributions, measures of central tendency and measures of variability were used to present and interpret the data in a meaningful way. Data cleaning was performed where data erroneously captured by the CHWs, PHC audiologists and/or the researcher, such as dates, were corrected to be in a uniform format. In cases where data was accidentally not captured by the researcher, the data collection forms were re-examined to supplement any missing data. Because the data differed significantly from normality (Shapiro–Wilk *p* < 0.05), nonparametric tests were used (Field, 2018). The Wilcoxon signed-rank (*W*) test was used to compare significant differences between dependent groups (baseline assessment and exit assessment). The Mann–Whitney U (*U*) test was used to determine whether there was a difference in the variables (timing of baseline assessments and the number of monitoring assessments attended by patients) for independent groups (patients assessed by CHWs and patients assessed by PHC audiologists). In order to determine significant predictors for hearing deterioration, multiple linear regression models with many assumptions were run initially; one of these assumptions is that the error terms must be normally distributed. The error terms in this study were not normally distributed. Therefore, quantile regression models, which are robust to outliers and do not require the assumptions of normally distributed error terms, were used instead. For inferential statistics, a 5% level of significance was used throughout.

The OMP used paper data collection forms, which were manually completed by the CHWs and PHC audiologists for each patient. Where important data were missing, this was because data were not recorded on the data collection forms by the testers, and were therefore unavailable for inclusion in this retrospective study.

### Ethical considerations

The study was conducted according to the guidelines of the Declaration of Helsinki and was approved by the Institutional Review Board (or Ethics Committee) of the University of Pretoria (GW20161128HS; 63/2017), the CoCT (7788) and the Western Cape Department of Health (WC_2017RP22_896). Owing to the retrospective nature of this study, consent to access the existing data collection forms on behalf of the patients was granted by the Western Cape Department of Health and the CoCT Health Department. All patient identifying information was kept confidential as patient records were given a numerical code in order to ensure anonymity during data collection and analysis. Data from the data collection forms were recorded on a password-protected Excel spreadsheet for later analysis by the researchers.

## Results

### Participants

Of the 831 patients included in the parent study, 201 met the participant selection criteria and were eligible for inclusion in this study. Seven patients with results indicating technical or procedural issues related to their baseline and exit assessment audiograms (i.e. improved thresholds [> 50 dB HL] across all frequencies) were excluded. A final sample of 194 patients (Figure 1) was included, as presented in Table 1. The mean age of patients was 36.2 years (standard deviation [SD] = 11.3; range = 15.0 – 65.1 years). The gender of 33.0% (64/194) of the patients was not recorded by CHWs and PHC audiologists on the data collection forms and was thus unavailable for inclusion in this retrospective study. At the time of the baseline assessment, 24.7% (48/194) of patients reported having DRTB and HIV co-infection, 20.6% (40/194) reported a history of excessive noise exposure and 18.0% (35/194) reported experiencing tinnitus. Patients’ baseline assessments were conducted, on average, 16.8 days (SD = 86.5; range = –494 to 14 days) before treatment initiation.

**TABLE 1:** Participant description at the time of the baseline assessment (*n* = 194).

Variables	%	*N*
**Gender**		
Not recorded	33.0	64
Male	35.6	69
Female	31.4	61
**Risk factor for ototoxicity**		
DRTB and HIV co-infection	24.7	48
Noise exposure	20.6	40
**Audiological symptoms**		
Tinnitus	18.0	35
Otalgia	6.2	12
Hearing loss	5.2	10
**Tester**		
CHW	76.3	148
PHC audiologist	23.7	46

DRTB, drug-resistant tuberculosis; HIV, human immunodeficiency virus; CHW, community health worker; PHC, primary healthcare.

### Ototoxicity monitoring programme characteristics

#### Timing and frequency of ototoxicity monitoring assessments

**FIGURE 2:**
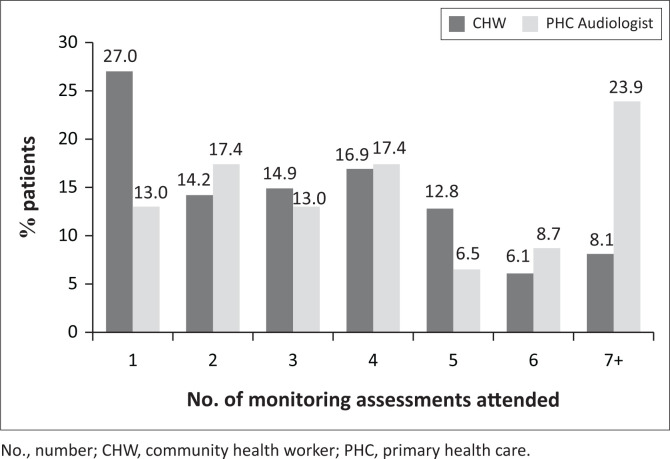
Percentage of patients attending assessments following the baseline assessment according to tester.

Community health workers tested 76.3% (148/194) of the patients in the study. There was a statistically significant difference (*p* = 0.003; *U* = 2406.5) between the timing of baseline assessments by CHWs and by PHC audiologists. Patients assessed by PHC audiologists had a baseline assessment conducted on average 52.0 days (SD = 134.9) before treatment initiation, while the patients assessed by CHWs had a baseline assessment conducted on average 5.9 days (SD = 61.2) before treatment initiation. There was a statistically significant (*p* = 0.019; *U* = 2637.0) difference between the average number of follow-up visits made by patients between CHWs and PHC audiologists; excluding the baseline assessment, patients assessed by PHC audiologists attended, on average, 4.3 (SD = 2.5; 46/194) monitoring assessments, while patients assessed by CHWs attended, on average, 3.3 (SD = 2.1; 148/194) monitoring assessments. Only 14.2% (21/148) of patients assessed by CHWs attended six or more monitoring assessments as recommended by the HPCSA (HPCSA, 2018) and the OMP protocol (Figure 2), compared with 32.6% (15/46) of patients assessed by PHC audiologists.

### Ototoxicity monitoring programme follow-up rates

The follow-up rates of the first six monitoring assessments for patients assessed by PHC audiologists (69.2% – 87.0%) were higher than for those assessed by CHWs (51.2% – 80.6%) (Table 2). The average days elapsed between monitoring assessments were fewer for patients assessed by CHWs (53.4 days; SD = 10.3) than for patients assessed by PHC audiologists (64.3 days; SD = 19.3) (Table 2). Both groups exceeded the 14–30 days between monitoring assessments recommended by the HPCSA (HPCSA, 2018) and the OMP protocol.

**TABLE 2:** Follow-up return rates and average days between consecutive pure tone audiometry assessments according to tester type.

Tester/Monitoring assessments	CHWs				PHC audiologists			
	Follow-up rate (%)	*n*/group total	Ave No. of days betweenassessments	SD	Follow-up rate (%)	*n*/group total	Ave No. of days betweenassessments	SD
1st – 2nd	73.0	108/148	47.8	37.8	87.0	40/46	68.1	83.9
2nd – 3rd	80.6	87/108	53.2	62.1	80.0	32/40	55.2	52.6
3rd – 4th	74.7	65/87	63.4	75.4	81.3	26/32	48.4	45.4
4th – 5th	63.1	41/65	52.3	44.3	69.2	18/26	87.8	107.3
5th – 6th	51.2	21/41	49.7	42.1	83.3	15/18	65.7	37.8

SD, standard deviation; CHW, community health worker; PHC, primary health care; Ave no., average number.

### Ototoxicity characteristics

#### Treatment effects on hearing

**FIGURE 3:**
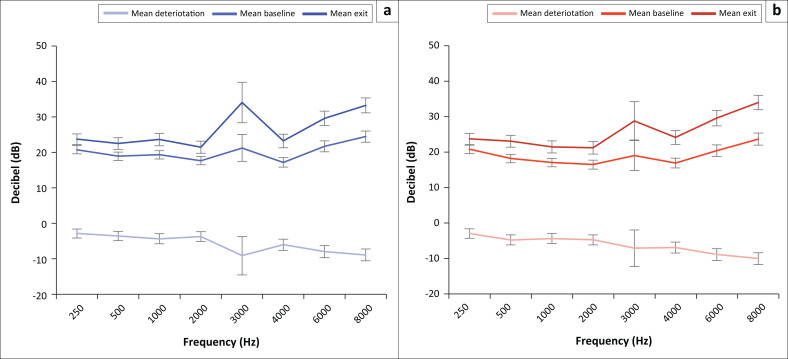
Mean hearing thresholds and deterioration of the left (a) and right (b) ears from baseline to exit assessment (*n* = 194) (error bars = standard error).

More than half (51.5%; 100/194) of the patients presented with a pre-existing hearing loss at the time of the baseline assessment, where a hearing loss was defined as one or more hearing threshold > 25dB HL in one or both ears across all frequencies, increasing to 66.5% (129/194) at the time of the exit assessment. On average, a decline in hearing thresholds from the baseline to exit assessment was evident across all frequencies bilaterally, with the deterioration most pronounced at the high frequencies (Figure 3 and Table 3). The mean hearing threshold deterioration was statistically significant at the high frequencies 4 kHz (*p* = 0.006; *W* = −2.744), 6 kHz (*p* < 0.001; *W* = −3.897) and 8 kHz (*p* < 0.001; *W* = −4.371) of the left ear, and at the frequencies 500 Hz (*p* = 0.021; *W* = −2.309), 1 kHz (*p* = 0.029; *W* = −2.178), 2 kHz (*p* = 0.005; *W* = −2.248), 4 kHz (*p* < 0.001; *W* = −3.573), 6 kHz (*p* < 0.001; *W* = −4.456) and 8 kHz (*p* < 0.001, *W* = −5.322) in the right ear (Table 3).

**TABLE 3:** Mean baseline and exit assessment hearing threshold values and hearing deterioration for the left and right ears (*n* = 194).

Frequency (Hz)	Mean baseline dB	SD	*n*	Mean exit dB	SD	*n*	Mean deterioration dB	SD	*n*
**Left ear**									
250	20.8	16.8	194	23.7	21.4	193	−2.8	17.8	193
500	18.9	16.6	194	22.5	23.0	193	−3.5	18.2	193
1000	19.3	16.2	194	23.7	24.3	194	−4.3	20.1	194
2000	17.7	16.4	194	21.5	23.8	193	−3.7	19.3	193
3000	21.3	16.9	20	34.1	29.4	27	−9.1	22.2	17
4000	17.2	18.6	194	23.2	26.1	194	−6.0	21.9*	194
6000	21.7	20.1	170	29.6	27.0	179	−8.0	22.2*	168
8000	24.5	21.9	194	33.3	29.0	193	−8.9	23.5*	193
**Right ear**									
250	20.8	16.2	192	23.7	22.1	193	−2.9	18.4	191
500	18.2	16.3	193	23.1	23.1	193	−4.8	19.7*	192
1000	17.0	16.1	193	21.5	23.6	194	−4.4	19.5*	193
2000	16.5	17.1	193	21.2	24.8	194	−4.7	20.0*	193
3000	19.0	18.9	20	28.8	27.1	25	−7.1	21.3	17
4000	16.9	19.2	193	24.1	28.1	194	−6.9	21.9*	193
6000	20.4	21.1	169	29.6	28.6	182	−8.9	21.8*	167
8000	23.7	23.3	193	34.0	28.7	194	−10.0	23.0*	193

Hz, Hertz; dB, decibel; SD, standard deviation.

* , statistical significance of *p* < 0.05.

**FIGURE 4:**
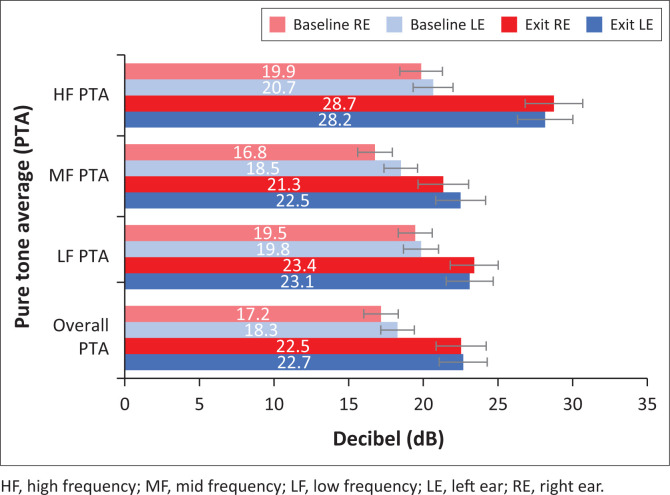
Mean baseline and exit assessment pure tone averages of the left and right ears (*n* = 194) (error bars = standard error).

The patients’ hearing thresholds were compared according to various pure tone averages (PTAs) in Table 4 as follows: overall PTA (0.5 kHz – 4 kHz), low-frequency PTA (0.25 and 0.5 kHz), mid frequency PTA (1 and 2 kHz) and high frequency PTA (3 kHz – 8 kHz). Hearing deterioration was evident across all PTA groups bilaterally; however, deterioration was most pronounced at high frequencies (Table 4 and Figure 4). The results indicated statistically significant deterioration in the mean high-frequency PTA for the left (*p* < 0.001; *W* = −4.125) and right (*p* < 0.001; *W* = −5.247) ears. The mean overall PTA deterioration (*p* = 0.001; *W* = −3.426) and the mid-frequency PTA deterioration (*p* = 0.017; *W* = −2.381) of the right ear from baseline to exit assessment was statistically significant (Table 4). There was no statistically significant difference in the mean hearing threshold deterioration at each frequency (*p* > 0.05; *W* = −1.499 – 0.240) or mean PTA deterioration between the left and right ears (*p* > 0.05; *W* = –1.675–0.637).

**TABLE 4:** Mean pure tone average values and hearing deterioration for the left and right ears (*n* = 194).

PTA frequency range (Hz)	Mean baseline dB	SD	*n*	Mean exit dB	SD	*n*	Mean deterioration dB	SD	*n*
Left ear									
Overall PTA (500–4000)	18.3	15.6	194	22.7	22.5	194	−4.4	17.6	194
LF PTA (250–500)	19.8	16.3	194	23.1	21.8	193	−3.2	17.2	193
MF PTA (1000–2000)	18.5	15.7	194	22.5	23.3	194	−4.0	18.6	194
HF PTA (3000–8000)	20.7	18.6	194	28.2	25.8	194	−7.6	20.6*	194
Right ear									
Overall PTA (500–4000)	17.2	16.0	193	22.5	23.3	194	−5.3	18.6*	193
LF PTA (250–500)	19.5	15.9	193	23.4	22.3	193	−3.9	18.4	192
MF PTA (1000–2000)	16.8	16.1	193	21.3	23.6	194	−4.5	19.0*	193
HF PTA (3000–8000)	19.9	19.8	193	28.7	26.8	194	−8.5	20.3*	193

PTA, pure tone average; Hz, Hertz; LF PTA, low-frequency pure tone average; MF PTA, mid-frequency pure tone average; HF PTA, high-frequency pure tone average; dB, decibel; SD, standard deviation.

* , statistical significance of *p* < 0.05.

### Description of ototoxic hearing loss

The presence of an ototoxic shift was determined according to the three criteria developed by ASHA (1994), the most widely used and validated criteria (AAA, 2009), as indicated in Table 5. Following DRTB treatment, more than half of the patients (51.5%; 100/194) presented with a significant ototoxic shift meeting one or more of the ASHA criteria. Ototoxic shifts occurred most often at high frequencies (4 kHz – 8 kHz). There was no statistically significant difference (*p* = 0.114; *W* = −1.581) in ototoxic shifts (meeting one or more of the ASHA criteria) between the left and the right ears.

**TABLE 5:** Distribution of patients presenting with an ototoxic shift at the time of exit assessment.

ASHA ototoxic shift criteria	No ototoxic shift evident %	*n*/194	Group 1† %	*n*/194	Group 2† %	*n*/194	Group 3† %	*n*/194
Patients	48.5	94	42.3	82	43.3	84	4.1	8
Left ear	62.9	122	32.5	63	29.4	57	2.1	4
Right ear	55.2	107	33.0	64	36.1	70	4.1	8
Bilateral (left and right)	70.6	137	23.2	45	22.2	43	2.1	4

*Source*: American Speech-Language-Hearing Association (ASHA). (1994). Audiologic management of individuals receiving cochleotoxic drug therapy. Retrieved from https://www.asha.org/policy/GL1994-00003/

ASHA, American Speech-Language-Hearing Association.

Group 1 shift of ≥ 20 dB at a single frequency; Group 2, shift of ≥10 dB at 2 adjacent frequencies; Group 3, shift to ‘no response’ at three consecutive frequencies.

† , 100/194 patients presented with an ototoxic shift that may have met one or more ASHA criteria: 16.0% (31) met one ASHA criterion, 33.0% (64) met two ASHA criteria and 2.6% (5) met three ASHA criteria.

The prevalence of hearing loss severity according to the revised WHO grades of hearing impairment is presented in Table 6. From the baseline to the exit assessment, the prevalence of patients presenting with hearing loss meeting any category of hearing loss severity increased from 22.2% (39/194) to 25.8% (50/194). For the left and right ears, from the baseline to the exit assessment, the prevalence of patients presenting with hearing loss meeting any category of hearing loss severity increased from 32% (62/194) to 39.7% (77/194), and from 27.3% (52/193) to 33.5% (66/193), respectively. Following DRTB treatment with kanamycin, there was an increase in patients presenting with a hearing loss meeting each category of hearing loss, excepting mild hearing loss, most notably for the moderate (4.9%; 9/194), total (1.5%; 3/194) and unilateral (9.8%; 19/194) categories of hearing loss severity.

**TABLE 6:** Prevalence of hearing loss severity for the left (*n* = 194) and right (*n* = 193) ears at the baseline and the exit assessment according to the revised World Health Organization grades of hearing loss.

Category	Patients†, ‡				Left ear‡				Right ear‡			
	Baseline		Exit		Baseline		Exit		Baseline		Exit	
	%	*n*/194	%	*n*/194	%	*n*/194	%	*n*/194	%	*n*/193	%	*n*/193
Normal hearing (-10 dB HL – 19.9 dB HL)	77.8	151	74.2	144	68	132	60.3	117	72.7	141	66.5	127
Mild hearing loss (20.0 dB HL – 34.9 dB HL)	19.1	37	15.5	30	23.7	46	23.2	45	17.5	34	16.5	32
Moderate hearing loss (35.0 dB HL – 49.9 dB HL)	1.5	3	4.6	9	4.1	8	5.7	11	3.6	7	8.2	16
Moderately severe hearing loss (50.0 dB HL – 64.9 dB HL)	1.0	2	2.1	4	1.5	3	5.2	10	2.1	4	1.5	3
Severe hearing loss (65.0 dB HL – 79.9 dB HL)	0.5	1	1.0	2	1.0	2	0.5	1	1.6	3	2.6	5
Profound hearing loss (80.0 dB HL – 94.9 dB HL)	0.0	0	1.0	2	0.5	1	2.1	4	2.1	4	2.6	5
Total hearing loss (≥ 95.0 dB HL)	0.0	0	1.5	3	1.0	2	3.1	6	0.0	0	3.1	6
Unilateral hearing loss (< 20.0 dB HL in the better ear, ≥ 35. 0 dB HL in the worse ear)	3.1	6	9.8	19	-	-	-	-	-	-	-	-

*Source*: Olusanya, B.O., Davis, A.C., & Hoffman, H.J. (2019). Hearing loss grades and the international classification of functioning, disability and health. *Bulletin of the World Health Organisation, 97*(10), 725–728. https://doi.org/10.2471/BLT.19.230367

db HL, decibel hearing level.

† , In the better ear.

‡ , Pure tone average of 500, 1000, 2000 and 4000 Hz.

### Predictors of hearing loss

The presence of a pre-existing hearing loss at the time of the baseline assessment was a significant predictor of the deterioration of the overall PTA (0.5 kHz – 4 kHz) of the left (β = −8.750; 95% confidence interval [CI] [−14.953; −2.547]; *p* = 0.006) and right (β = −13.750; 95% CI [−19.063; −8.437]; *p* < 0.001) ears over time. Patients presenting with a pre-existing hearing loss had an increase in deterioration of 8.75 and 13.75 times more for the left and right ear, respectively, than those with no pre-existing hearing loss. A history of noise exposure was a second significant predictor of the deterioration for overall PTA (0.5 kHz – 4 kHz) of the right ear (β = −3.750; 95% CI [−6.682; −0.818]; *p* = 0.012), with patients who indicated exposure to noise having an increase in deterioration of 3.75 times more than those with no history of exposure to noise. Significant predictors of the deterioration of the high frequency PTA (3 kHz – 8 kHz) of the right ear, where hearing deterioration was most prominent, included DRTB and HIV co-infection (β = −5.833; 95% CI [−10.711; −0.956]; *p* = 0.019) and the presence of a pre-existing hearing loss (β = −26.667; 95% CI [−35.521; −17.812]; *p* < 0.001). Quantile regression models showed that gender, age, duration of administration of medication, history of tinnitus and tester (CHW or PHC audiologist) were not significant predictors of hearing deterioration (*p* > 0.05).

## Discussion

### Ototoxicity monitoring programme characteristics

#### Community health workers as facilitators of decentralised community-based ototoxicity monitoring

The majority (76.3%) of patients in this study were assessed by CHWs, possibly because there were more CHWs (six) acting as testers than PHC audiologists (two). The follow-up rates between consecutive monitoring assessments for patients assessed by CHWs reached as high as 80.6%. In addition, the average number of days between assessments was lower for patients assessed by CHWs (53.4 days) than for those assessed by PHC audiologists (64.3 days). The follow-up rate of patients assessed by CHWs is better than the rate of a community-based DRTB treatment programme that included ototoxicity monitoring, where the loss to follow-up was reported as being high as 38% (Moyo et al., 2015). Increased usage of ototoxicity monitoring and DRTB treatment services has been associated with older age (> 26 years) (Moyo et al., 2015), timing of baseline assessments (within 1 month of treatment initiation), the presence of pre-existing hearing loss and the development of ototoxic hearing loss following treatment (Ramma, Nhokwara, & Rogers, 2019). The high follow-up rate (80.6%) and the shorter timing between monitoring assessments (53.4 days) for patients assessed by CHWs in the present study may therefore be attributed to the average age of patients (36.2 years), the timing of baseline assessments (5.9 days before treatment duration), the high prevalence (51.5%) of pre-existing hearing loss and the development of ototoxic hearing loss in patients (51.5%) according to one or more of the ASHA criteria following DRTB treatment. In addition, the decentralised community-based nature of the OMP, offered in a PHC framework of care and integrated into DRTB treatments services for increased patient access to care may have attributed to the higher follow-up rates and timing between ototoxicity monitoring assessments for patients assessed by CHWs evident in this study.

Numerous challenges to the implementation of ototoxicity monitoring exist, including a shortage of trained health care professionals and a lack of resources to conduct serial monitoring (Dillard et al., 2021; Khoza-Shangase & Masondo, 2021). Sub-Saharan Africa has an extremely low coverage of ear, nose and throat, audiology and speech therapy services, and the availability of equipment remains poor (Mulwafu et al., 2017). The COVID-19 pandemic has had a severely negative impact on the provision and access to TB services for patients in many countries (Migliori et al., 2021; WHO, 2020c, 2021a, 2021b) exacerbating the existing challenges in treating TB and monitoring associated with ototoxicity. World Health Organization guidelines promote the establishment of community-based TB services primarily delivered by CHWs, and in the context of the COVID-19 pandemic such programmes may mitigate the additional strain on healthcare services and the delivery of essential TB services (WHO, 2020a), including ototoxicity monitoring. The employment of CHW for community-based hearing screening has been shown to provide increased access to hearing services (Bright et al., 2019; Eksteen et al., 2019; Mulwafu et al., 2017) and may offer a solution to the shortage of human resources synonymous with ototoxicity monitoring in South Africa (O’Donovan, Verkerk, Winters, Chadha, & Bhutta, 2019). In addition, integrating ototoxicity monitoring into existing community-based DRTB treatment services in PHC allows for a patient-centred approach that can increase patients’ access to services (Cox et al., 2014). The findings of the current study indicating high follow-up rates and shorter number of days between assessments for patients assessed by CHWs, support the feasibility of a community-based model of care for ototoxicity monitoring facilitated by CHWs as a widely used service.

#### Frequency and timing of ototoxicity monitoring assessments and ototoxicity monitoring programmes data management procedures

Although there were positive outcomes for community-based ototoxicity monitoring facilitated by CHWs, the OMP failed to meet some quality benchmarks pertaining to the frequency and timing of ototoxicity monitoring assessments, as stated in the guidelines (HPCSA, 2018) and the OMP protocol. A few patients (14.2% – 32.6%) were assessed with the regularity required by the OMP protocol and the HPCSA. Ideally, ototoxicity monitoring should be conducted every 14 (HPCSA, 2018) to 30 days (OMP protocol); however, the OMP was unable to assess patients with the frequency recommended, with assessments being conducted, on average every 2 months or more (53.4–64.3 days). This demonstrates a missed opportunity for the early detection of hearing loss that could support preventative actions through a change in treatment regimens (Crundwell, Gomersall, & Baguley, 2016). A previous report has also indicated that audiologists conducting ototoxicity monitoring in South Africa do not conduct monitoring assessments with the frequency recommended by the national guidelines (Khoza-Shangase & Masondo, 2020).

Significant differences in the frequency of ototoxicity monitoring by CHWs and by PHC audiologists were identified in this study. The number of monitoring assessments attended by patients assessed by PHC audiologists (mean 4.3) was higher than for those assessed by CHWs (mean 3.3). In addition, almost a third (32.6%) of patients assessed by PHC audiologists attended the recommended six follow-up assessments, compared to 14.2% of patients assessed by CHWs. The reasons for patients assessed by PHC audiologists attending monitoring assessments with more frequency than those assessed by CHWs could not be established in this study. However, a possible reason may be the supervision and quality control provided by OMP managers of ototoxicity monitoring conducted by CHWs. For CHWs to fulfil their role successfully, regular training and supervision are required (WHO, 2007). Reports from sub-Saharan Africa indicate that the current provision of training for CHWs is not sufficient to improve the quality of care in this region (O’Donovan, O’Donovan, Kuhn, Sachs, & Winters, 2018). Possible suggestions to facilitate ongoing training and supervision for CHWs include the use of tools such as smartphone technology and applications like WhatsApp (O’Donovan et al., 2018). In addition to ototoxicity monitoring for DRTB, CHWs were tasked with conducting home-based, school-based and PHC clinic-based hearing screening services at various locations across the Mitchells Plain/Klipfontein sub-district. This may have affected the ability of CHWs to visit PHC and community health clinics with the frequency required to conduct regular ototoxicity monitoring assessments. Furthermore, patient retention to services during the long and arduous DRTB treatment regimen is known to be difficult, even in a well-resourced programme (Moyo et al., 2015; Ramma et al., 2019); however, the patient variables influencing the frequency of ototoxicity monitoring assessments could not be determined because of the retrospective nature of this study.

The current OMP used paper data collection forms that were manually completed by CHWs and PHC audiologists. However, important demographic information, such as patient gender (33%), was not recorded by CHWs and PHC audiologists, and was, therefore, unavailable for analysis owing to the retrospective nature of this study. An effective OMP data management system enables the comparison of serial monitoring through reliable data recording; this is more efficiently achieved using an electronic data management system (Khoza-Shangase & Masondo, 2021). The use of smartphone technology and cloud-based data management has been shown to offer effective data management for large-scale screening purposes (Eksteen et al., 2019) and is recommended for South African OMPs. Integrating secure data sharing with national health repositories should be considered in an effort to improve the data management procedures of OMPs in South Africa (Swanepoel, 2020).

### Ototoxicity characteristics

#### Treatment effects on hearing and predictors of hearing loss

In resource-limited countries such as South Africa, baseline audiometric assessments are often not conducted within the recommended timeframe, before ototoxic damage is likely to occur (Ganesan et al., 2018; Govender & Paken, 2015; Khoza-Shangase & Masondo, 2020) and pre-existing hearing loss is consequently underdiagnosed (Hong et al., 2020). As a result, there are limited data on the prevalence of pre-existing hearing loss in DRTB patients, apart from a recent study by Hong et al. (2020). In the current study, where patients had a baseline assessment conducted on average 16.8 days before treatment initiation, more than half (51.5%) presented with a pre-existing hearing loss. The findings of this study support recently published reports that found that pre-existing hearing loss is prevalent in South African DRTB patients, with 60% of patients assessed using conventional audiometry presenting with a pre-existing hearing loss prior to treatment (Hong et al., 2020). The prevalence of pre-existing hearing loss is an important consideration for South African OMPs, as patients presenting with a pre-existing hearing loss prior to DRTB treatment initiation are at particular risk of developing further hearing loss following the use of aminoglycoside (Hong et al., 2020; Petersen & Rogers, 2015). The increased risk of aminoglycoside-induced hearing loss in DRTB patients with a pre-existing hearing loss is confirmed by the results of the current study, which indicated that patients presenting with a pre-existing hearing loss at the time of the baseline assessment had an increase in hearing deterioration up to 13.75 times higher than those with no pre-existing hearing loss.

A history of noise exposure was a significant predictor of hearing deterioration in the current study, with patients who reported previous exposure to noise presenting with 3.75 times the deterioration in hearing sensitivity compared with those with no history of noise exposure. A previous report concurred, indicating that patients with a history of noise exposure and aminoglycoside treatment had poorer high-frequency hearing thresholds than those exposed to noise without a history of aminoglycoside treatment (Khoza-Shangase, 2020). The findings of this study emphasise the importance of counselling for DRTB patients so that they avoid excessive noise exposure during and after aminoglycoside treatment (Campbell & Le Prell, 2018). In addition, where hearing deterioration was most prominent at the high frequencies (3 kHz–8 kHz), a significant predictor of hearing loss was DRTB and HIV co-infection. The current study supports previous findings that HIV-infected DRTB patients are more likely to develop an aminoglycoside-induced hearing loss than their non-infected peers (Harris et al., 2012; Hong, Budhathoki, & Farley, 2018). Several significant predictors of hearing loss in DRTB patients were observed, including the presence of a pre-existing hearing loss, HIV co-infection, and a history of exposure to noise. The findings of the current study have important implications for OMPs as they highlight the need for OMPs to identify and prioritise DRTB patients presenting with pre-existing hearing loss, HIV co-infection, and noise exposure for all-oral treatment regimens, together with more vigilant audiological ototoxicity monitoring for early management of hearing deterioration. Patients presenting with these conditions should be identified by OMPs for closer supervision of attendance of ototoxicity monitoring assessments, through direct communication with patients using, for example, smartphone technology and applications like WhatsApp.

#### Description of hearing loss

The reported prevalence of ototoxicity varies widely and depends on various factors, including drug type and dosage, and patients’ demographic profile, such as age (> 60 years), the presence of mitochondrial mutations and exposure to loud noises (Ramma et al., 2019). In addition, a lack of standardised research methodology and the use of different criteria to grade and classify hearing loss has influenced the estimates of hearing loss prevalence (Campbell & Le Prell, 2018; Dillard et al., 2021; Ganesan et al., 2018). In the current study, it was found that following DRTB treatment with kanamycin, more than half of the patients (51.5%) presented with a significant ototoxic shift meeting one or more of the ASHA criteria, with ototoxic shifts most often occurring at high frequencies. The finding of this study concurs with a recent hearing loss prevalence estimation, using ASHA criteria, of 49.7% following kanamycin use (Dillard et al., 2021). At the time of the baseline assessment, 51.5% of patients in the current study presented with one of more elevated hearing thresholds (> 25dB HL) in one or both ears across all frequencies; this increased to 66.5% (129/194) at the time of the exit assessment. In order to report on the severity of hearing loss following DRTB treatment among the patients in this study, and to describe the functional consequences for communication associated with each category of severity, the prevalence of hearing loss severity was presented according to the revised WHO grades of hearing impairment. In the current study, following DRTB treatment with kanamycin, there was a notable increase in patients presenting with hearing loss meeting the moderate (4.9%), total (1.5%) and unilateral (9.8%) categories of hearing loss severity (Olusanya et al., 2019; WHO, 2021b). Patients with untreated moderate or unilateral hearing loss following DRTB treatment may experience difficulties hearing speech in the presence of background noise, while patients presenting with total hearing loss will be profoundly deaf, resulting in a devastating impact on the quality of life (WHO, 2021b).

Patients in this study presented with a bilateral decline in hearing thresholds in all PTA groups, with the most pronounced deterioration at high frequencies at the time of the exit assessment. Drug-resistant TB treatment using kanamycin therefore had a negative effect on the hearing status of the patients in this study, with clinically and statistically significant deterioration of hearing thresholds, most markedly in the high frequencies. The findings of the current study, therefore, support the implementation of OMPs for DRTB patients who are administered aminoglycosides, particularly as the latest WHO DRTB treatment guidelines (WHO, 2020b) continue to include amikacin, which is known to be ototoxic. The occurrence of high-frequency hearing deterioration measured in this study further supports the recommendation (HPCSA, 2018) of the use of EHF audiometry for ototoxicity monitoring in DRTB patients, particularly for those most at risk for developing ototoxic hearing loss. Extended high-frequency audiometry, which assesses air conduction hearing thresholds above 8 kHz, is considered to be the most sensitive behavioural method for detecting early cochlear outer hair cell damage (Campbell & Le Prell, 2018; Harris, Peer, & Fagan, 2012; Petersen & Rogers, 2015) before it affects hearing functionality, and is therefore recommended for the monitoring of ototoxicity (HPCSA, 2018). There have been significant advances in point of care testing and mobile health technologies in hearing assessment (Garinis et al., 2021), which should be considered for ototoxicity monitoring in South Africa. In particular, the use of smartphone technology with automated EHF audiometry hearing assessment applications and cloud-based capabilities for integrated data management should be considered for community-based ototoxicity monitoring (Bornman, Swanepoel, De Jager, & Eikelboom, 2019; Eksteen et al., 2019; WHO, 2021b; Yousuf Hussein, Swanepoel, Mahomed, & Biagio de Jager, 2018).

The limitations of this study included the absence of quality indicators for audiometry conducted by CHWs and PHC audiologists. In addition, the prevalence of adverse side effects experienced by patients was not established by testers at the time of exit assessment. Immittance measures were not included as part of OMP protocol, and therefore, the prevalence of ototoxic hearing loss may have been influenced by the inclusion of patients presenting with middle-ear disorders. Important data pertaining to patient description and treatment were at times not recorded by testers and were, therefore, unavailable for inclusion in this retrospective study, and thus, may have caused research bias. Researcher and analysis triangulation were applied to reduce the effects of research bias. The use of a non-probability sampling method may limit the generalisability of the results of this study.

## Conclusion

The findings of this study support the employment of CHWs to facilitate community-based ototoxicity monitoring of patients with DRTB. However, the findings reveal that over time, community-based OMPs for DRTB show gaps in service delivery practices, most notably in the frequency and timing of ototoxicity monitoring assessments. The possible reasons for this may highlight the need for ongoing training and supervision of CHWs using novel tools, such as smartphone technology and applications like WhatsApp. Drug-resistant TB treatment with kanamycin caused clinically and statistically significant deterioration of hearing thresholds in patients, most prominently at high frequencies. In this study, the patients co-infected with HIV, those with a pre-existing hearing loss and those exposed to excessive noise were at higher risk for developing ototoxicity-induced hearing deterioration. Patients presenting with these conditions should be identified and prioritised by OMPs for more vigilant ototoxicity monitoring and all-oral treatment regimens. South African OMPs need support and novel approaches for community-based ototoxicity monitoring, with revision of the current recommendations to best suit the South African context. These may include the widespread integration of ototoxicity monitoring services facilitated by CHWs into the existing decentralised, community-based PHC service delivery frameworks using a portable, automated technology with integrated data-sharing capabilities.
